# Lessons Learned from CDC’s Global COVID-19 Early Warning and Response Surveillance System

**DOI:** 10.3201/eid2813.212544

**Published:** 2022-12

**Authors:** Philip M. Ricks, Gibril J. Njie, Fatimah S. Dawood, Amy E. Blain, Alison Winstead, Adebola Popoola, Cynthia Jones, Chaoyang Li, James Fuller, Puneet Anantharam, Natalie Olson, Allison Taylor Walker, Matthew Biggerstaff, Barbara J. Marston, Ray R. Arthur, Sarah D. Bennett, Ronald L. Moolenaar

**Affiliations:** Centers for Disease Control and Prevention, Atlanta, Georgia, USA (P.M. Ricks, G.J. Njie, F.S. Dawood, A.E. Blain, A. Winstead, A. Popoola, C. Li, J. Fuller, P. Anantharam, N. Olson, A. Taylor Walker, M. Biggerstaff, B.J. Marston, R.R. Arthur, S.D. Bennett, R.L. Moolenaar);; Agency for Toxic Substances and Disease Registry, Atlanta (C. Jones)

**Keywords:** COVID-19, respiratory infections, severe acute respiratory syndrome coronavirus 2, SARS-CoV-2, SARS, coronavirus disease, zoonoses, viruses, coronavirus, public health surveillance, validation study, Centers for Disease Control and Prevention, United States, World Health Organization, Early Warning and Response Surveillance

## Abstract

Early warning and response surveillance (EWARS) systems were widely used during the early COVID-19 response. Evaluating the effectiveness of EWARS systems is critical to ensuring global health security. We describe the Centers for Disease Control and Prevention (CDC) global COVID-19 EWARS (CDC EWARS) system and the resources CDC used to gather, manage, and analyze publicly available data during the prepandemic period. We evaluated data quality and validity by measuring reporting completeness and compared these with data from Johns Hopkins University, the European Centre for Disease Prevention and Control, and indicator-based data from the World Health Organization. CDC EWARS was integral in guiding CDC’s early COVID-19 response but was labor-intensive and became less informative as case-level data decreased and the pandemic evolved. However, CDC EWARS data were similar to those reported by other organizations, confirming the validity of each system and suggesting collaboration could improve EWARS systems during future pandemics.

On December 31, 2019, newspapers in China reported a cluster of 27 pneumonia cases of unknown etiology in Wuhan and noted concern for the re-emergence of severe acute respiratory syndrome (SARS) coronavirus (*1*), which caused a global outbreak of respiratory illness during 2002**–**2003 (*2*). On January 13, 2020, the novel respiratory illness now known as COVID-19 was detected outside of China. By May 13, 2022, a total of 517,648,631 confirmed COVID-19 cases and 6,261,708 deaths had been reported from 231 countries, territories, and locations (*3*). 

In response to the COVID-19 outbreak, the US Centers for Disease Control and Prevention (CDC) activated its emergency operations center on January 20, 2020, to direct CDC’s domestic and international preparedness and response efforts. The breadth and speed of COVID-19’s spread presented considerable challenges to global early warning and response (EWAR), for which the objective is early detection of public health events that require rapid investigation and response (*4*). EWAR incorporates 2 different surveillance systems, indicator-based surveillance (IBS) and event-based surveillance (EBS). IBS is the systematic collection, monitoring, analysis, and interpretation of structured data (i.e., indicators), produced by numerous identified, predominantly, health-based formal sources (*4*). IBS data are not used solely for EWAR purposes, but are collected for other surveillance objectives, such as measuring impact of programs or identifying priority health problems (*4*). However, IBS systems are often constrained by reporting delays and limited surveillance capacity. These constraints led the World Health Organization (WHO), through its International Health Regulations (IHR), to encourage member states to build and strengthen their IBS and EBS capacities as part of EWAR systems for public health threats (*5*). 

EBS is the organized collection, monitoring, assessment, and interpretation of mainly unstructured, ad hoc information regarding health events or risks that could represent an acute threat to human health (*4*). EBS is a functional component of EWAR. The information collected for EBS is diverse and originates from multiple, often unpredetermined sources, both official and unofficial, including rumors reported by the media or ad hoc reports from informal networks. The information collection process is mainly active and conducted through a systematic framework specifically established for EBS purposes (*4*). IBS and EBS are complementary systems within EWAR, but EBS is used more frequently (Figure 1) (*4*,*6*).

**Figure 1:**
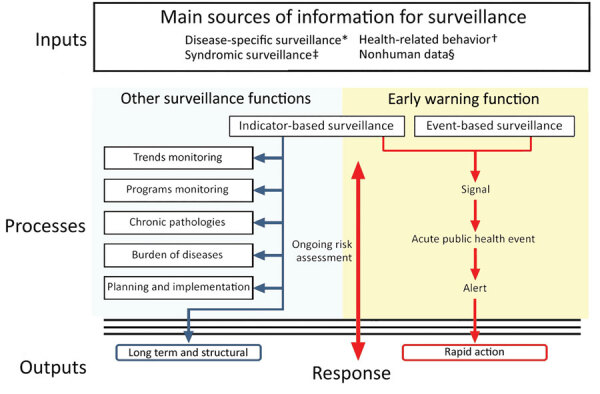
Overview of public health surveillance and response functions used in an evaluation of the Centers for Disease Control and Prevention Early Warning and Response Surveillance system. Adapted from the World Health Organization (*4*). *Conventional human surveillance based on biological confirmation of cases.†Human case data based on syndromic definition. ‡Data and information in relation to human health (e.g., media reports, sick leave, medicine sales, population movement, social unrest, etc.). §Veterinary surveillance (zoonosis), environmental or biological surveillance (e.g., meteorlogical, vector density, water and air quality, etc.).

As part of CDC’s response to COVID-19, the agency implemented the CDC global COVID-19 Early Warning and Response Surveillance (CDC EWARS) system to collect, process, analyze, interpret, and disseminate data about COVID-19 cases and deaths that occurred outside of the United States. In contrast to CDC EWARS, WHO’s IBS system is considered the benchmark for international surveillance data, because its IBS is based on direct reporting of case-level data from national health authorities (*7*). However, several other institutions also established global surveillance systems to monitor the COVID-19 epidemic during the prepandemic phase, including the Johns Hopkins University (JHU) Center for Systems Science and Engineering and the European Centre for Disease Prevention and Control (ECDC) (*8*,*9*). The COVID-19 pandemic is occurring in an era of crowdsourcing—defined as engaging a large group of persons to rapidly gather data (*10*)—an approach used by JHU. We describe CDC EWARS during the prepandemic period, January 20–March 7, 2020, and its use to guide evidence-based decisions. To validate CDC EWARS case, death, and affected country counts, we compared them to counts reported by WHO; to assess the consistency of CDC EWARS counts, we compared them with counts reported by JHU and ECDC.

## Methods

### Description of CDC EWARS

CDC EWARS was established to collect data on all laboratory-confirmed COVID-19 cases reported outside the United States. Formal information sources included press statements and situation reports from ministries of health, national public health institutions, laboratory networks, and WHO. Informal sources included media reports; social media feeds; the data aggregator Epidemic Intelligence in Open Source (*11*); and information shared by email from partners, CDC colleagues, and CDC’s 59 country offices. Informal reports of suspected or confirmed COVID-19 cases and deaths were verified as confirmed cases or deaths by using official websites and other official social media platforms, including Twitter (https://www.twitter.com), Facebook (https://www.facebook.com), and Instagram (https://www.instagram.com). We downloaded and archived source documents. Surveillance activities were conducted daily, including weekends, from 8:00 AM to 11:59 PM Eastern Time.

**Figure 2:**
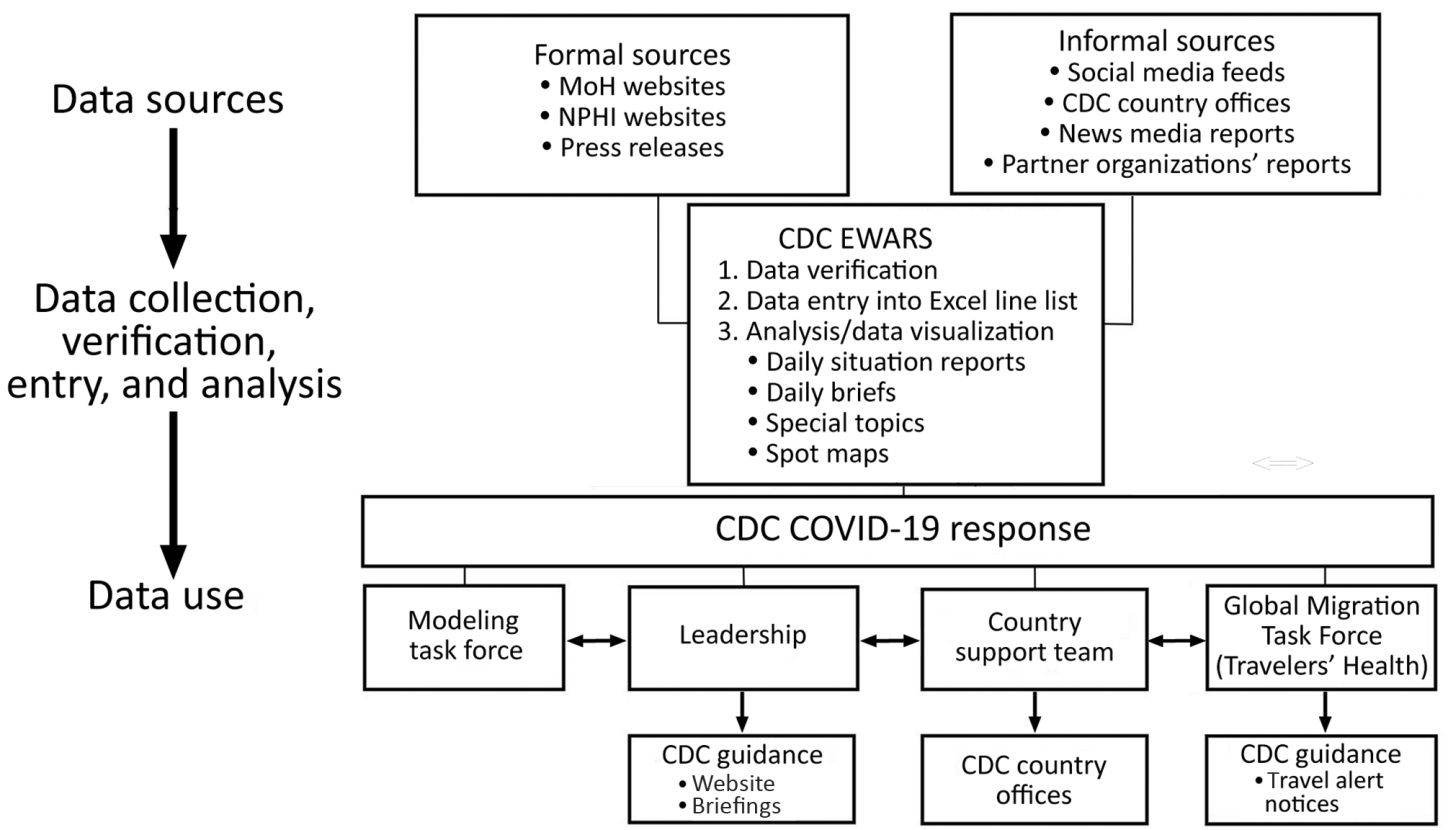
Work and information flow for CDC EWARS during epidemiologic weeks 3–9, January 20–March 7, 2020. CDC EWARS, US Centers for Disease Control and Prevention global COVID-19 Early Warning and Response Surveillance system; MoH, ministry of health; NPHI, national public health institutions.

We recorded the daily COVID-19 data for officially confirmed cases and deaths in narrative format and abstracted these into an Excel spreadsheet (Microsoft, https://www.microsoft.com) to create a case line list (Figure 2). Any variable lacking an explicit affirmative or negative narrative statement was coded as missing. Because mainland China data were in aggregate, those data were not included in the line list. The global case line list data were available for analyses each weekday morning, including data entered up to midnight the preceding day, and were maintained through epidemiologic week (EW) 9, ending March 7, 2020. Deaths often were reported in aggregate; therefore, we maintained data on country aggregate death counts in a separate spreadsheet through EW 8, after which we used WHO death counts. The case line list included 57 variables, encompassing demographic, case detection management (e.g., hospitalization and isolation), clinical, and exposure information data.

### Data Collection Methods for Other Surveillance Systems

We identified 3 additional daily sources for global COVID-19 case, death, and country count data: WHO, JHU, and ECDC (Table 1). WHO collects IBS data in accordance with the IHR (*12*), under which member states submit daily laboratory-confirmed COVID-19 case-level data to WHO by using a standardized case reporting form or line list, following WHO technical guidance on COVID-19 surveillance (*13*). However, on February 27, 2020, WHO recognized that reporting case-level data was not always feasible and provided explicit guidelines for submission of aggregate daily incidence and deaths and weekly submission of aggregate data on other demographic, clinical, and exposure information (*14*). WHO daily COVID-19 situation reports were published in the late afternoon Eastern Time, and data were current as of 5:00 AM Eastern Time. We downloaded daily situation report data for these analyses on March 2 and March 7, 2020 (*15*,*16*).

**Table 1:** Comparison of surveillance methodology among the 4 global COVID-19 surveillance systems used in an evaluation of CDC’s global COVID-19 EWARS system*

Methodology	CDC EWARS	WHO IBS	JHU IBS and EBS	ECDC EWAR
Only report on confirmed cases and deaths	Y	Y	Y	Y
Case-level data	Y	Y	N	Y
Data cutoff time	11:59 PM ET	5 AM ET	Evening	5 AM ET
Reporting time	Morning, next day	Afternoon, same day	Evening, same day	Afternoon, same day

*CDC, Centers for Disease Control and Prevention; EBS, event-based surveillance; ECDC, European Centres for Disease Control; EWAR, early warning and response; EWARS, Early Warning and Response System; IBS, indicator-based surveillance; JHU, Johns Hopkins University Center for Systems Science and Engineering; WHO, World Health Organization.

The JHU dashboard began online publication on January 22, 2020, to provide real-time data on laboratory-confirmed case (WHO definition), death, and recovery counts in affected countries. The JHU system started with morning and evening manual data collections from various sources, but on February 1, 2020, JHU migrated the system to a semi-automated living system strategy that included manual updates throughout the day (*8*). JHU collected data from Twitter feeds, online news services, and direct crowdsource communications sent through the dashboard. Data were verified manually by using case counts from official country and international sources. For comparative analysis, we downloaded JHU data from a Github repository on March 22, 2020 (*17*).

ECDC collected data during 1:00–5:00 AM Eastern Time for its daily COVID-19 situation reports, following a standard process (*9*). ECDC data comprised IBS and EWAR data submitted by health agencies in Europe and international partners, complemented by information from official government websites and official social media accounts. ECDC also screened several unofficial media and social media sources, but ECDC only aggregated confirmed cases and deaths reported by the national and regional authorities for their database. ECDC’s daily COVID-19 situation reports were published in the afternoon Eastern Time (*18*), along with a copy of the database of daily case and death counts. We downloaded ECDC data for our analyses on March 19, 2020.

### Descriptive Methods for CDC EWARS

We describe the personnel and person-hours needed to develop and maintain CDC EWARS for EW 3, ending January 25 (the first week of COVID-19 CDC EOC activation) through EW 9, ending March 7, 2020. We also describe data provided to CDC leadership from the line list’s daily analyses and use of the line list by other response teams for decision-making. We examine data completeness by assessing the percentage of nonmissing data for selected variables.

### Analytic Methods to Assess Validity and Consistency of CDC EWARS Data

To assess the validity of case, death, and country count data collected through CDC EWARS, we compared the weekly cumulative counts during EW 3–9 to counts reported by WHO; to assess consistency, we compared the weekly cumulative counts to counts from JHU and ECDC. For all comparisons, we excluded data for mainland China and the United States because those data were obtained from different sources by the different systems. We also performed head-to-head comparisons of CDC EWARS data to data from the other 3 systems by subtracting CDC EWARS weekly cumulative country case counts from those reported in the 3 other systems and examining scatter plots of the differences. Because CDC and JHU implemented surveillance aimed at providing the most up-to-date information, we also compared dates of report for each country’s first case. We analyzed data in SAS version 9.4 (SAS Institute, https://www.sas.com). This activity was reviewed by CDC and was conducted consistent with CDC policy and applicable federal law, including 45 CFR part 46.102(l)(2); 21 CFR part 56; 42 USC §241(d); 5 USC §552a; and 44 USC §3501.

## Results

### Person-Time and Expertise Required to Implement and Maintain CDC EWARS

The CDC EWARS team was formed January 20, 2020, starting with 1 person and eventually expanding to a 7-person team (Table 2); all members had at least a master’s degree. The team’s growth coincided with the increase in global case counts and increased number of countries reporting cases. Team members worked an average of 8.2 hours/day, 7 days/week (Table 2), but various team members still worked considerable overtime (i.e., >40 hours/week), from 5–45 hours of overtime per person per week. The weekly total person-hours increased from 70 in EW 3 to 345 in EW 9, for a 7-day workweek; 1,726 person-hours were required to develop and maintain the CDC EWARS system.

**Table 2:** Hours worked by CDC EWARS team during COVID-19 epidemiologic weeks 3–9, January 20–March 7, 2020*

Indicator	Epidemiologic week; beginning date							Total
	3; Jan 25	4; Feb 1	5; Feb 8	6; Feb 15	7; Feb 22	8; Feb 29	9; Mar 7	
No. team members	1	4	5	6	7	7	7	9†
Average no. hours worked/d‡	10.0	10.2	9.2	7.6	6.7	6.8	7.0	8.2
Total person-hours/wk	70	184	294	235	296	302	345	1,726
Cumulative no. reporting countries	13	25	28	29	32	63	103	–
No. new cases	38	135	186	331	1,037	5,238	17,346	24,311

*Data are based on a 7-day work week. CDC, US Centers for Disease Control and Prevention; EWARS, early warning and response surveillance; –, not applicable. †The team comprised 9 different persons during study period. ‡Accounts for the average no. hours each person worked per day during the week.

### Application of CDC EWARS Data 

Data from the CDC EWARS were used to develop daily internal and high-level situation reports and spot maps. Situation reports included global, regional, or country-specific cumulative and incident case and death counts, epidemic curves, analyses of case exposure and case demographic characteristics, and identification and description of geographic spread, clusters, and transmission chains (*19*). The CDC EWARS team provided daily information to CDC leadership to identify countries at risk, prioritize support for at-risk countries, and assess importation risk to the United States. Moreover, the team also provided these reports and data to the 59 CDC country offices and other response teams for situational awareness, which informed additional analyses and preparedness and response activities.

CDC’s COVID-19 Response Modeling Team used the line list data from CDC EWARS to estimate the preliminary case fatality ratios outside mainland China; provide estimates of the incubation period and time-to-death; and evaluate the risk for COVID-19 importation to the United States and other countries. These analyses contributed to the early understanding of the basic epidemiology of COVID-19, informed risk assessments, and helped identify geographic areas that might be at greater risk for COVID-19 introduction and transmission (*20*).

Daily data from the CDC EWARS line list were also pivotal to determining the alert level for travel health notices that were posted during the study period (*21*). Information used included increases in the number of cases in a short period, geographic distribution of cases, evidence of sustained (multi-generational) transmission, transmission chains, and international exportations. The information also was used to inform targeted risk assessment and public health management of arriving international travelers.

### Data Quality

#### Completeness of Data Collection

By March 7, 2020, CDC EWARS had detected 24,311 confirmed cases and 405 deaths globally. Analysis of exposure patterns revealed that 100% of weekly cases had exposure information in EW 3 and 87% had information in EW 5 (Table 3). However, as case counts began increasing in EW 6, countries provided less information on exposure; by EW 9, only 1.9% of cases had an exposure determination (Table 3). Data also were incomplete for other variables. During the first week of the epidemic, the 2 variables with the most complete data were age (87%) and sex (100%), but both variables decreased to <2% completeness at EW 9, by which time all variables had <2% completeness (Table 2). 

**Table 3:** Data completeness collected by CDC EWARS system for selected variables during epidemiologic weeks 3 thru 9, January 20–March 7, 2020*

Variables	Epidemiologic week						
	3	4	5	6	7	8	9
Total new cases	38	135	186	331	1,037	5,238	17,346
Patient demographics							
Age	33 (86.8)	106 (78.5)	87 (46.8)	97 (29.3)	156 (15)	156 (3)	325 (1.9)
Sex	38 (100)	115 (85.2)	91 (48.9)	97 (29.3)	157 (15.1)	190 (3.6)	291 (1.7)
Nationality	37 (97.4)	66 (48.9)	54 (29)	52 (15.7)	48 (4.6)	82 (1.6)	121 (0.7)
Place of residence	27 (71.1)	39 (28.9)	28 (15.1)	17 (5.1)	61 (5.9)	161 (3.1)	47 (0.3)
Clinical indicators							
Date of illness onset	16 (42.1)	74 (54.8)	58 (31.2)	59 (17.8)	57 (5.5)	60 (1.2)	36 (0.2)
Date person sought care	20 (52.6)	67 (49.6)	58 (31.2)	45 (13.6)	13 (1.3)	13 (0.3)	8 (0.1)
Fever	21 (55.3)	46 (34.1)	45 (24.2)	32 (9.7)	29 (2.8)	28 (0.5)	27 (0.2)
Cough	12 (31.6)	33 (24.4)	34 (18.3)	27 (8.2)	20 (1.9)	30 (0.6)	15 (0.1)
Exposures							
Travel, China	38 (100)	126 (93.3)	95 (51.1)	77 (23.3)	59 (5.7)	63 (1.2)	27 (0.2)
Travel, excluding China	1 (2.6)	20 (14.8)	46 (24.7)	43 (13)	34 (3.3)	190 (3.6)	297 (1.7)
Contact with confirmed COVID-19 case	15 (39.5)	65 (48.2)	142 (76.3)	153 (46.2)	386 (37.2)	135 (2.6)	74 (0.4)
Any exposure information†	38 (100)	129 (95.6)	161 (86.6)	163 (49.2)	389 (37.5)	274 (5.2)	336 (1.9)
First exposure date	3 (7.9)	10 (7.4)	13 (7)	6 (1.8)	0	0	0
Last exposure date	18 (47.4)	63 (46.7)	26 (14)	16 (4.8)	0	1 (<0.001)	0

*Values are no. (%) of total new cases per week. Total data points collected, n = 24,311. CDC, US Centers for Disease Control and Prevention; EWARS, Early Warning and Response Surveillance. †Includes any information regarding travel or contact with a confirmed case.

#### Validity and Consistency among Surveillance Systems

**Figure 3:**
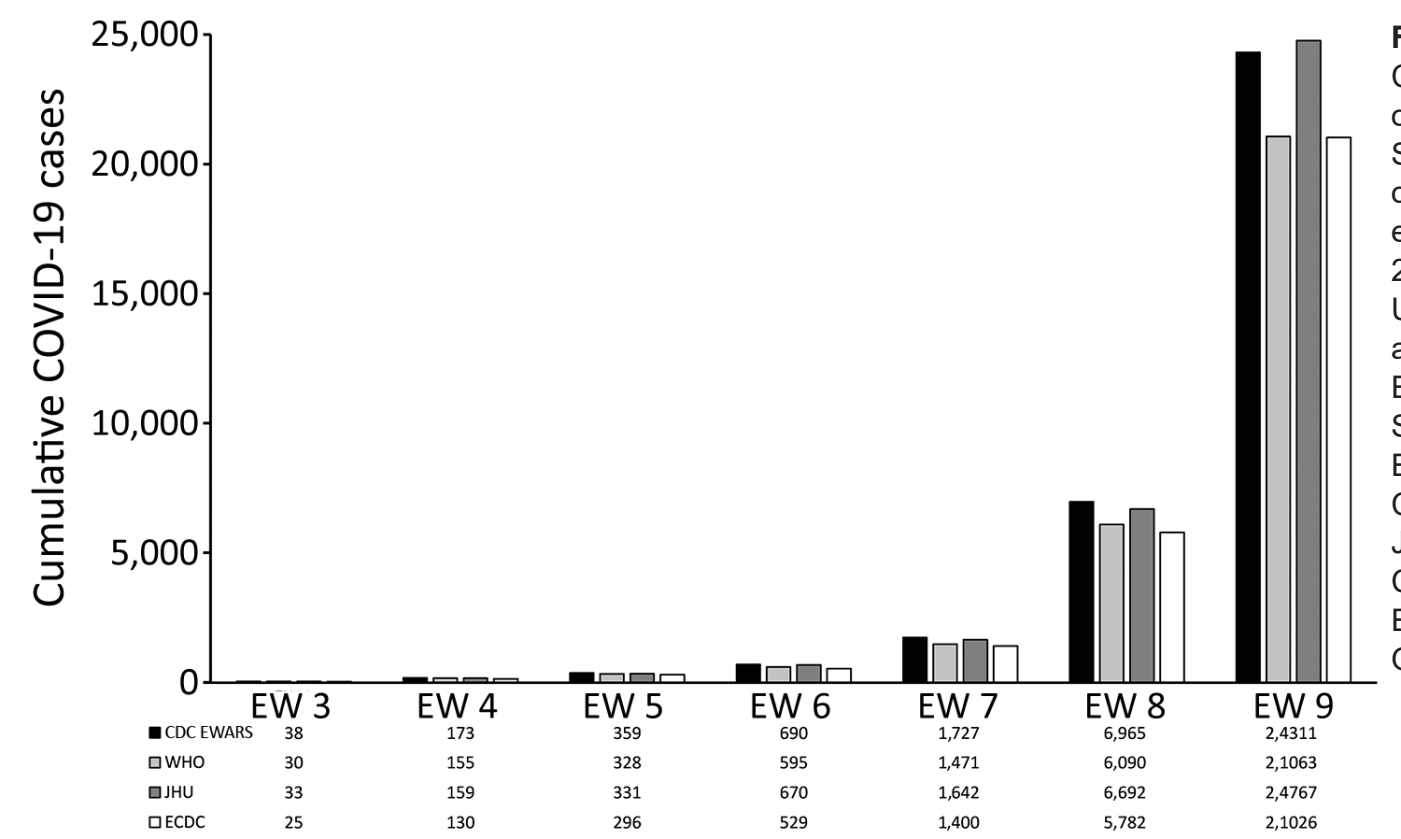
Cumulative confirmed COVID-19 cases reported outside of mainland China and the United States by CDC EWARS and other surveillance systems during epidemiologic weeks 3–9, January 20–March 7, 2020. CDC EWARS, US Centers for Disease Control and Prevention global COVID-19 Early Warning and Response Surveillance system; ECDC, European Centers for Disease Control; EW, epidemiologic week; JHU, Johns Hopkins University Center for Systems Science and Engineering; WHO, World Health Organization.

**Figure 4:**
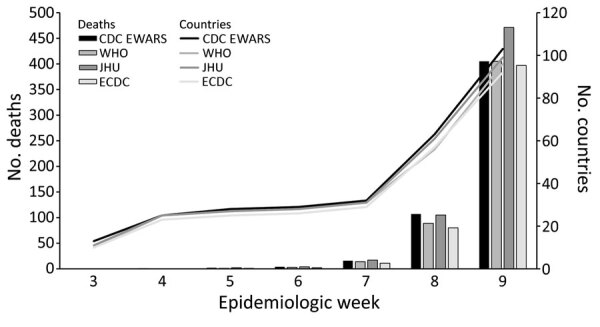
Cumulative reported confirmed COVID-19 deaths and cumulative number of countries reporting confirmed COVID-19 cases for CDC EWARS, JHU, WHO, and ECDC systems during epidemiologic weeks 3–9, January 20–March 7, 2020. WHO death counts were used as CDC EWARS inputs after epidemiologic week 8. Scales for the y-axes differ substantially to provide data on 2 different indicators and are not intended for direct comparisons. CDC EWARS, US Centers for Disease Control and Prevention global COVID-19 Early Warning and Response Surveillance system; ECDC, European Centers for Disease Control; JHU, Johns Hopkins University Center for Systems Science and Engineering; WHO, World Health Organization.

By the end of EW 9, March 7, 2020, COVID-19 cases had been reported from 104 countries, excluding mainland China and the United States, across the 4 surveillance systems. At the end of EW 9, the total reported confirmed cases reported by CDC was 24,311; by WHO was 21,063; by JHU was 24,767; and by ECDC was 21,026 (Figure 3). The 4 different surveillance systems all recorded the same general trend in cumulative cases across EW 3–9 (Figure 3). However, whereas CDC and JHU case counts were similar, WHO and ECDC case counts were close to one another but lower than those for CDC and JHU. The number of reported deaths and reporting countries described by the 4 systems was initially similar but diverged in EW 8 (Figure 4).

**Figure 5:**
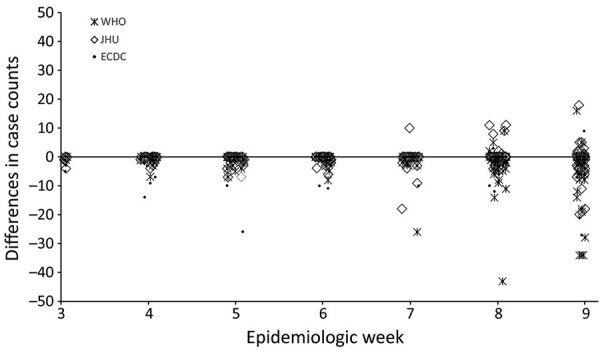
Scatterplot showing differences in individual country COVID-19 cumulative case-counts outside of mainland China and the United States between JHU, WHO, or ECDC systems, and CDC EWARS system during epidemiologic weeks 3–9, January 20–March 7, 2020. A value of zero indicates CDC EWARS and the other system had the same number of weekly cumulative cases for a given country; a negative value means that CDC EWARS reported a higher number of cases; and a positive value means that the other surveillance system reported more cases than CDC EWARS. Differences of >50 cases between CDC EWARS and WHO or ECDC for cumulative country case counts occurred in 6% (18/295) of instances, and between CDC EWARS and JHU in 1% (4/295) of instances. CDC EWARS, US Centers for Disease Control and Prevention global COVID-19 Early Warning and Response Surveillance system; ECDC, European Centers for Disease Control; JHU, Johns Hopkins University Center for Systems Science and Engineering; WHO, World Health Organization.

Agreement between CDC EWARS and the other 3 systems decreased over time, and dispersion of differences increased as the outbreak progressed and the case numbers rapidly rose (Figure 5). We also noted decreased agreement between JHU and WHO and between JHU and ECDC but noted less disagreement between JHU and CDC EWARS (data not shown). Differences of >50 cases between CDC EWARS and WHO or ECDC for cumulative country case counts occurred in 6% (18/295) of instances during the study period, primarily in countries with rapid increases in case counts during EW 7–9, which sometimes resulted in multiple daily updates. Differences of >50 cases between CDC EWARS and JHU occurred in only 1% (4/295) of instances. In identifying new countries reporting cases, CDC EWARS and JHU both reported the same date for 67% (70/104) of new countries; JHU reported an earlier date for 5% (5/104) and CDC EWARS reported an earlier date for 28% (29/104), of which 4 countries reported cases before JHU began its reporting.

## Discussion

CDC EWARS data were used to inform the agency’s international response activities, modeling efforts, travel health notice decisions, and manage arriving international travelers. When validated against data from WHO, CDC EWARS reported similar case, death, and country counts and was consistent with data from JHU and ECDC for most epidemiologic weeks assessed. The similarity of results between CDC EWARS and JHU also supports JHU’s finding of comparable case counts between its system and WHO and the validity of real-time data reporting on the JHU dashboard (*8*). Most higher counts reported by CDC EWARS were likely the result of different cutoff times for data collection and the different time zones of reporting countries (Table 1), which was compounded for WHO by the lag in reporting through the IHR mechanism.

The primary objective of EWARS is early detection of unusual events that might indicate an outbreak and enable a rapid response; however, in the context of an epidemic or pandemic, timely, valid, and useful systems to inform decision-making are even more imperative. In line with this consideration, CDC EWARS was most useful in the early phase of the epidemic, when case counts were relatively small and detailed data were publicly available to help address the many unanswered questions. The system was useful for providing broad overviews of the global situation but also flexible enough to target specific country and regional issues to inform CDC guidance and travel health notices, which are a critical CDC function during international outbreaks.

Data collection by multiple systems might be redundant and inefficient in the context of a rapidly developing pandemic, but each system’s objectives might differ. The JHU’s primary objective was to develop a public-facing interface that tracked COVID-19 cases, deaths, and recoveries, and it was a crucial public source for up-to-date information. CDC EWARS, however, was an internal system used to clarify the epidemiology of COVID-19 and thus help determine the agency’s international and domestic response. Although CDC EWARS contained official, publicly available data on confirmed cases and deaths, analyses of these data were not disseminated publicly, perhaps representing a missed opportunity to provide information to the public and to demonstrate transparency regarding the basis for certain policy decisions. However, other sources were available for these data. For instance, WHO and ECDC reported aggregate data on age and sex, and these data were officially provided by member countries. For CDC, making this information public would have required additional validation steps, resources, and clearances that were not in place during the early phase of the pandemic. Although providing more data to the public could be valuable, its usefulness and effects are more difficult to judge because of the large amount of missing data among the additional variables on which CDC could have reported and the intercountry variability of data completeness and comparability. In addition, providing yet another data source with different numbers could be confusing. CDC’s new Data Modernization Initiative could put the agaency in a stronger position to collect and report early surveillance data in the future.

The first limitation of the CDC EWARS system is that it was based on publicly available data, so content for some of the variables collected, especially clinical information, might be less accurate than medical records. Second, detailed reporting of COVID-19 cases by official sources declined as countries began to report more cases. Thus, data completeness in later weeks was low relative to earlier weeks, and data for age, which usually had high completeness, was <50% in the third week of data collection. Third, data reported for each case was not standardized, and a bias toward recording positive statements might have been introduced, leaving negative responses missing from the narrative. Finally, death counts were often provided in aggregate and could not be attributed to specific patients in the line list, thus precluding case-level analyses using death as the outcome.

The main lessons learned in implementing CDC EWARS were related to human resources, monitoring, and evaluation. The numbers of cases and affected countries made CDC EWARS labor-intensive. Because of the need to collect data from multiple time zones, expanding staffing to provide 24-hour shift coverage and surge capacity at system start-up would have been helpful. We found it necessary to evaluate the surveillance system as the outbreak progressed. By frequently monitoring the level of missing information and staff workload, we were able to discontinue the CDC EWARS system after EW 9 and transition the team to using official data from WHO and China to monitor aggregate non-US case and death counts. In retrospect, we could reasonably have discontinued CDC EWARS or greatly reduced the number of data collection variables after EW 6. However, limited knowledge of the novel agent at the time led us to continue CDC EWARS for a few additional weeks. After EW 9, we reduced the global line list to only 13 countries, which we selected on the basis of the quality of data, regional relevance, and potential impact on the United States. During the same period, the unfeasibility of case-based surveillance led WHO to continue to require countries to report daily case and deaths counts but to only require weekly aggregate reporting of case-level characteristics.

In conclusion, CDC EWARS was a useful tool for timely elucidation of the epidemiology and geographic distribution of COVID-19 and helped inform US response decisions and priorities, including travel health notices. The system was most useful in the early weeks of the epidemic, when case-level data were needed and available, enabling analysis of transmission dynamics, incubation period, and levels of community transmission. However, the evolution of an epidemic into a pandemic limits an organization’s ability to sustain case-level global EWARS beyond the early weeks. EWARS systems can still be useful at national and regional levels for early detection of events and timely decision-making, but global EWARS systems are most effective when countries publicly share data about critical variables on a structured, timely, and ongoing basis. The comparable incidence and mortality data found in our analysis across the 4 different surveillance systems indicated that future strategic collaboration among global systems could help leverage resources and reduce redundancies, particularly for longer-term surveillance. Such practices could enable different surveillance systems to expand their scopes to include other factors, such as interventions and their effectiveness, so that countries can quickly share best practices and other systems could focus on rapid reporting of fewer but more highly referenced variables.
